# COVID-19 in patients with chronic myeloid leukaemia on tyrosine kinase inhibitor therapy: a Honduran observational study

**DOI:** 10.3332/ecancer.2022.1481

**Published:** 2022-12-02

**Authors:** Carlos J Fajardoa, Eda Sofía Cálixb, Rafael Mojicac, Flora Duarted

**Affiliations:** Centro De Cáncer Emma Romero De Callejas (CCERC), Tegucigalpa 11101, Honduras; a https://orcid.org/0000-0002-6850-8308; b https://orcid.org/0000-0001-8700-8803; c https://orcid.org/0000-0003-2582-8851; d https://orcid.org/0000-0002-9707-9864

**Keywords:** Leukaemia, myeloid, chronic, BCR-ABL positive, COVID-19, imatinib mesylate

## Abstract

**Introduction:**

In the earliest cases of COVID-19, a higher percentage of severe and fatal cases was observed in patients with cancer, including those with haematological malignancies. However, patients with chronic myeloid leukaemia (CML) had better prognoses, suggesting that tyrosine kinase inhibitors (TKIs) may have a therapeutic effect against SARS-CoV-2. This study describes the clinical and epidemiological characteristics of patients with CML receiving the TKIs tested for SARS-CoV-2 in Tegucigalpa, Honduras.

**Methodology:**

An *Analytical cross-sectional* study was conducted. The sample included patients with Philadelphia chromosome-positive (Ph+) CML, who had been tested at least once for COVID-19 at the Emma Romero de Callejas Cancer Centre (CCERC). Sociodemographic and clinical variables were both analysed. Epi Info 7.2.4.0 and Stata/MP 16.0 were used to collect and analyse data. The COVID-19 positivity percentage and the association between severity and the TKI used were determined using Fisher’s exact test and odds ratio (OR). Data were gathered from clinical records with approval of CCERC institutional management.

**Results:**

One hundred and forty-nine patients with Ph+ CML were included; 20.1% were COVID-19-positive; 56% were male; mean age was 46 years; 81% were receiving imatinib, with a mean treatment duration of 6 years; 55% achieved a BCR -ABL molecular response ≤ 0.1% (IS). Twenty-one percent had comorbidities. COVID-19 was asymptomatic in 38.7% of patients, mild in 35.5% and severe in 9.7%. One patient died, a fatality rate of 3.2%. No statistical association was found between disease severity and treatment with imatinib versus second-line TKI (OR: 0.833, *p*: 0.8493, 95% CI: 0.098–10.998).

**Conclusion:**

Despite high COVID-19 positivity in CML when compared with the literature, this study found a lower fatality rate. The type of TKI used or molecular response at the time of infection was not associated with case severity. Determining the effectiveness of imatinib or other TKIs as a COVID-19 treatment requires randomised clinical trials.

## Introduction

On 20 January 2020, the disease caused by the SARS-CoV-2 virus, COVID-19, was declared a global public health emergency owing to its rapid worldwide spread [1]. Evidence obtained from observational studies conducted throughout 2020 showed that people with underlying diseases had a greater risk of developing severe COVID-19. In contrast with the mortality rates of 1.33%–11.6% reported for people without malignant diseases (for September 2020), the *COVID-19 and Cancer Consortium* and a UK-led cohort study reported mortality rates in cancer patients of 16% and up to 35%, respectively [2, 3].

Information on the prevalence of COVID-19 in patients with malignant haematological diseases remains limited. But it is thought while these patients are not more likely to become infected with SARS-CoV-2, their risk of becoming seriously ill and therefore dying is greater [4]. However, this has not been true for chronic myeloid leukaemia (CML), a myeloproliferative neoplasm with *t*(9:22) chromosomal translocation that affects the peripheral blood and bone marrow alike. The presence of CML has not been reported as a risk factor for severe COVID-19 illness. The current oncological prognosis for patients with CML has improved significantly since the introduction of imatinib, a tyrosine kinase inhibitor (TKI). Imatinib inhibits the interaction between the BCR-ABL1 (BCR- ABL is a fusion gene also called Ph chromosome (BCR-ABL fusion gene). Formed by fusion of the 3´ sequences from ABL1 (Abelson) gene at 9q34 to the 5´ portion of the BCR (breakpoint cluster region) gene sequences at 22q11) oncoprotein and adenosine triphosphate, blocking the proliferation of malignant clones. Imatinib is so effective that patients with CML on TKIs have normal or near-normal lives, with a 10-year survival rate of roughly 90% [5]. A cross-sectional study conducted in Hubei found that the prevalence of COVID-19 in 551 patients with CML was 0.9%, compared with 0.1% in the general population [6]. Furthermore, an Italian study that asked treating physicians about the incidence of COVID-19 in patients with CML found it was extremely low in those taking TKIs (0.17%) [7]. In July 2020, the International CML Foundation (iCMLf) had found 91 cases of COVID-19 in patients with CML from 20 countries (85% of them identified through polymerase chain reaction (PCR) and/or serology testing), and estimated COVID-19 prevalence at 0.7% [8].

Imatinib is a weak-base drug that accumulates in cell lysosomes over 1,000-fold more than in the extracellular compartment. It has shown antiviral activity owing to the lysosomal alkalinisation required for virus–cell fusion. It is thus reasonable to assume that imatinib accumulated in airway epithelial cells could protect against SARS-CoV-2 infection [9]. Imatinib has also shown *in vitro* activity against SARS-CoV-2 infection [10]. A hypothesis has therefore been proposed that imatinib may protect against COVID-19 by blocking the fusion of SARS-CoV-2 S proteins with patient cells, thus preventing the endocytosis the virus requires [7]. Establishing this causality requires obtaining conclusive evidence through randomised clinical trials.

The objective of this study was to determine the clinical and epidemiological characteristics of patients with CML on TKI therapy, who had COVID-19 diagnostic testing at the Emma Romero de Callejas Cancer Centre (CCERC) between June 2020 and March 2021. Specifically, the sociodemographic factors and the COVID-19 positivity percentage in patients with CML were described. The association between the use of TKIs and the degree of COVID-19 severity and fatality rate was also determined.

## Methodology

An *analytical cross-sectional* study was conducted. The study population included CCERC patients with a Philadelphia chromosome-positive (Ph+) CML diagnosis confirmed by real-time quantitative PCR (RT-qPCR) testing for the BCR-ABL oncogene using GeneXpert equipment. These patients also had at least one SARS-CoV-2 test since diagnostic testing became available in June 2020, using one of the three main routine testing techniques: RT-PCR, antigen testing or antibody testing. Notably, following WHO COVID-19 diagnostic criteria (https://apps.who.int/iris/bitstream/handle/10665/336482/WHO-2019-nCoV-Surveillance_Case_Definition-2020.1-spa.pdf), the initial study plan was to perform SARS-CoV-2 serum antibody tests for all patients with CML willing to pay, regardless of whether they had COVID-19 symptoms. Positive results would then be confirmed using RT-PCR testing (the gold standard for COVID-19 diagnoses). Unfortunately, the economic status of patients participating in the TKI donation programme was precarious, and many patients who tested positive lacked funds to pay for the confirmatory RT-PCR test. However, the presence of antibodies indicates a person has been exposed to the COVID-19 causative virus. Monitoring antibody seropositivity in a given population thus makes it possible to infer serological prevalence and incidence [11]. On the basis of these facts and other studies of serological prevalence of COVID-19 in patients with CML, it was decided to include patients with positive antibody tests in the group of COVID-19-positive patients [12, 13]. RT-PCR could confirm this diagnosis in some patients, confirming active infection in only these patients at enrolment. The CCERC is a non-profit institution and lacks the funds to finance testing for patients. Patients receiving imatinib (Gleevec) have routine haematological evaluations every 4 months, and patients receiving nilotinib (Tasigna) or dasatinib (Sprycel) have these evaluations every 2 months.

A form-based data-collection tool was created to document CML and COVID-19 cases previously reported and validated by the iCMLf, which was also documenting cases globally for the CANDID study. Four of the cases included in this analysis were also included in the CANDID study database [8]. The data-collection tool included sociodemographic variables, personal history, description of the signs and symptoms suggesting COVID-19 at each visit, CML haematological and molecular status when conducting COVID-19 tests, type of test conducted and medical management if positive. Data collection began in June 2020 with the increased national availability of COVID-19 tests, and this study analysis included data up to March 2021. No patient had received any COVID-19 vaccine at the time of data collection.

Data were taken from medical records and entered into an Epi Info 7 template. The principal investigator was responsible for data entry quality, consistency checks and data-cleansing processes. The analysis was conducted using the STATA 15 statistical package. Normality tests were applied to numeric variables using the Shapiro–Wilk Test, which determined normal distribution of variables. After determining normality, the student’s *t*-test was used to calculate the mean and compare it between COVID-19-positive and COVID-19-negative patients with their corresponding 95% CI and *p*-value. The frequency, relative percentage and test of association of qualitative variables were calculated using the chi-squared test or Fisher’s exact test. The COVID-19 positivity percentage in patients with CML was determined. The association between the ordinal variable, which establishes COVID-19 severity, and the TKI used at the time of infection diagnosis, was calculated using Fisher’s exact test. Further, the strength of association between a severe/non-severe COVID-19 dichotomous variable and the use of imatinib versus other TKIs, was calculated using odds ratio (OR). The statistical association was calculated between the molecular response at the time of infection, measured by BCR-ABL RT-PCR, and infection severity in patients with COVID-19 was calculated.

Disease severity was classified in one of these groups: 1) Asymptomatic illness: patients who tested positive for SARS-CoV-2 using a virologic (PCR or antigen) or antibody test but showed no signs of illness consistent with COVID-19; 2) Mild illness: patients who had COVID-19 signs or symptoms (e.g. fever, cough, odynophagia, headache, general malaise, muscle pain, nausea, vomiting, diarrhoea, dysgeusia and/or anosmia), but did not have shortness of breath, dyspnoea or abnormal chest imaging; 3) Moderate illness: patients who showed evidence of lower respiratory disease (dyspnoea) during clinical or radiological assessment and who maintained an oxygen saturation ≥ 94% on room air at sea level; 4) Severe illness: patients with oxygen saturation < 94%, a ratio of arterial oxygen partial pressure to fractional inspired oxygen (PaO2/FiO2) < 300 mm Hg, a respiratory rate > 30 breaths per minute or lung infiltrates > 50% and 5) Critical illness: patients with respiratory failure, septic shock and/or multiple organ dysfunction. In patients with no available oxygen saturation, PaO2/FiO2 or radiological information, severity was classified on the basis of clinical manifestations and the level of medical care patients required (mild – at-home care, moderate – required supplemental oxygen at outpatient centres, severe – required hospital ward or intensive care unit admission). The illness was considered non-severe in patients with mild and asymptomatic illness, and severe in patients with moderate, severe and critical illness.

The study was approved by CCERC institutional management. Collecting clinical data only from medical records, without direct interaction with patients, ensured confidentiality. Ethical issues pertaining to database management were considered, but this protocol was not submitted to an ethics committee because study data sources were CCERC medical records.

## Results

This analysis included 149 patients with a Ph+ CML diagnosis, at least 20.1% of whom had COVID-19 (31/149). The distribution of positive test results by technique were (only testing to detect COVID-19): for rapid tests, 21 (67%); for rapid test + Enzyme-linked immunosorbent assay (ELISA), 1 (3%); for rapid test + RT-PCR, 4 (13%); for RT-PCR, 3 (10%); and for ELISA alone, 2 (6%). A majority of patients were male, 56% (83/149) (*p* = 0.055) and patient age at which COVID-19 was detected was 50.6 years (range: 16–74). The age of patients in whom COVID-19 was not detected was 44.7 years (range: 12–88) (*p* = 0.0747). Imatinib was the most common TKI used to treat CML in both groups (81%, 121/149), with no difference between the groups in the proportion of patients taking imatinib. Average TKI treatment duration was 65.7 months in the group with COVID-19 and 78.4 months in the group without COVID-19. There were no significant differences in the comparison of means. Fifty-five percent of patients (82/149) achieved a BCR-ABL molecular response of <0.1% [International Scale (IS)], 14% (21/149) achieved a response of >1%–10% (IS) and 22% (32/149) achieved a response >10% (IS). No statistical association was found between BCR-ABL molecular response and detection of COVID-19. The following comorbidities were found in the patients included in this study: hypertension, 13; diabetes mellitus, 9; asthma, 3; epilepsy, HIV, chronic kidney disease and heart disease, 1; no patients were obese. Fifteen percent of patients (23/149) had other comorbidities in addition to CML, and no statistical association was found between the two groups (*p* = 0.073). Patient demographic and clinical characteristics are in Table 1.

The most common clinical signs and symptoms in COVID-19-positive patients were fever (45.2%), muscle and joint pain (29.0%), cough (25.8%) and dyspnoea (25.8%) (Table 2). Breaking down infection severity, 38.7% of COVID-19-positive patients (12/31) had an asymptomatic illness, 35.5% (11/31) had a mild illness, 12.9% (4/31) had a moderate illness and 12.9% (4/31) had a severe illness. One patient died, a fatality rate of 3.2% (1/31). The deceased patient was a 65-year-old man recently diagnosed with CML who had received low-dose imatinib for 8 months and whose BCR-ABL was >10% (IS). This patient also had diabetes mellitus and chronic kidney disease, and needed haemodialysis. Table 3 shows the frequency of cases by severity versus TKI used at the time of infection. No statistical association was found between disease severity (severe versus non-severe) and treatment with imatinib versus second-line TKI (OR: 0.833 *p*-value: 0.8493, 95% CI: 0.098–10.998) (Table 4). There was also no statistical association between COVID-19 disease severity and BCR-ABL molecular response at the time of the study (Table 5).

## Discussion

This study found a COVID-19 positivity rate of 20% in a population of 149 patients diagnosed with CML between June 2020 and March 2021 at the CCERC in Tegucigalpa, Honduras. This proportion is notably higher than that found in a multicentre observational study that Pagnano *et al* [14] conducted in five Latin American countries (Brazil, Argentina, Chile, Peru and Mexico). That study found an incidence of 2.3% (92/3,933) and a case fatality rate of 11.9% (11/92), although in a much larger population of patients with CML. Case fatality in this study was 3.2%, while in the CANDID Study it was 14% (12/87) [8]. An Italian study using data from the Campus CML nationwide programme found an incidence of COVID-19 in patients with CML of 2.5% (217/8,665), a mortality rate of 0.13% and a case fatality rate of 5.5% (12/217) [15]. Differences in sample size might explain the dissimilar proportions, incidences and fatality rates seen among the mentioned studies. Mortality in patients with other haematologic malignancies is markedly higher, reaching 34% in a systematic review and meta-analysis of 3,377 cases [16].

Fifty-six percent of patients in this study were male, 70.9% (22/31) of patients with COVID patients were male and 51.7% (61/118) of patients without COVID-19 were male. But there was no statistical significance to these differences in proportions. This predominance of male patients is consistent with the multicentre Latin America study, which also found 56% of patients were male, and with a cross-sectional study conducted in China that characterised patients with COVID-19 and CML [6]. The Italian national study found an even higher percentage of males, 73% [15]. An observational study conducted in China between December 2019 and January 2020 in the general population found 41.9% of patients out of 1,099 cases from 552 hospitals were female [17].

The mean age of the study sample was 45.9 years, for patients with COVID-19 it was 50.6 years and for patients without COVID-19 it was 44.7 years. But there was no statistical significance to these differences in means. In the multicentre Latin American study of patients with CML, age at COVID-19 diagnosis was 48 years (range 22–79) [14], and in the Chinese study that analysed the general population with COVID-19, it was 47 years [17].

Eighty-one percent of the population of this study (121/149) were receiving imatinib (Gleevec), the first-line treatment for patients with CML. Thirteen percent of patients (19/149) were receiving nilotinib (Tasigna) and 6% (9/149) were receiving dasatinib (Sprycel). Bosutinib and ponatinib were available when these data were analysed, but no patients receiving these drugs met the study inclusion criteria. This finding differs from those of the CANDID study, which included 110 patients with CML and COVID-19, 36% of whom were receiving dasatinib, 17% imatinib and 2% nilotinib; in addition, 11% were receiving bosutinib and 1% ponatinib [8]. The time elapsed between CML diagnoses and COVID-19 testing was 75.7 months (65.7 months in patients with COVID-19 versus 78.4 months in patients without COVID-19, *p* = 0.223). This is consistent with the CANDID and Latin American multicentre studies, which reported that the mean times between diagnosis of CML and COVID-19 were 7 and 8 years, respectively [8, 14].

GeneXpert PCR testing for BCR-ABL molecular response found that IS percentage was <0.1% for 55.0% of patients, from 0.1%–1% for 9.4%, >1%–10% for 14.1% and >10% for 21.5%. In patients who tested positive for COVID, 51.6% had IS < 0.1%, with no significant differences between the proportions compared to the group without COVID-19. Moreover, no statistical association was found between the molecular response to TKIs and the severity of COVID-19. These data are consistent with those of Pagnano *et al* [14], who reported that 43.2% of patients had a molecular response of 4 or 4.5 and 25% had a major molecular response, representing approximately 70% of the sample analysed.

Twenty-one percent (21/149) of patients with CML in this study had at least one other comorbidity. Among patients with COVID-19, 26% (8/31) had some comorbidity, versus 13% (15/118) of those not diagnosed with COVID-19 (*p* = 0.073). Higher percentages of comorbidities were found in the Campus CML nationwide programme, which reported that 56% of COVID-19 and CML cases had other comorbidities, mainly hypertension, diabetes, dyslipidaemia and other cardiovascular disorders [15]. The Latin American multicentre study found that 42% of patients with CML and COVID-19 had other comorbidities [14].

COVID-19 was asymptomatic or mild in 77% (24/31) of patients, moderate in 13% (4/31) and severe in 10% (3/31). As a population parameter (patients without CML), a database from the Chinese Centre for Disease Control and Prevention presented 72,314 cases of COVID-19 through February 2020. Eighty-one percent of symptomatic cases were mild, 14% were severe and 5% were critical [18]. The CANDID study analysing patients with CML reported that COVID was asymptomatic in 7% of patients, mild in 45%, moderate in 17% and severe in 17% [8]. The Latin American study reported 9% of COVID-19 cases were asymptomatic, 53% were mild, 14% were moderate, 13% were severe and 13% were critical [14]. Percentages of mild COVID-19 were lower in CML populations than in non-CML populations, but mild remains the most common degree of severity. Our study found that COVID was mild in 38% of patients, but also a higher percentage of asymptomatic patients (38%) compared to other studies. Notably, all patients were tested at routine appointments even if they had no symptoms suggesting COVID-19, a practice that could explain the high percentage of asymptomatic cases. That finding is consistent with some studies that found percentages of asymptomatic cases ranging from 10.7% to 56.5% [19].

Additionally, when examining the severity of COVID-19, no statistically significant differences were found between the use of imatinib versus second-line TKIs. This finding is consistent with the findings that Bascı *et al* [20] reported for a retrospective observational study in Turkey that analysed 16 cases of patients with COVID-19 and CML. When the study groups were divided by the TKI they received (imatinib, nilotinib or dasatinib), no statistically significant differences were found between the proportions of intensive care unit admissions, the need for mechanical ventilation and the case fatality rate. In this study, the mortality rate in patients with CML and COVID-19 was 6.3% versus 12.8% in a non-CML control group (with a ratio of 3 controls per case) [20]. The CANDID study found the factors associated with elevated mortality included advanced age and the use of imatinib versus other TKIs (25% for imatinib versus 3% for a second-generation TKI, *p* = 0.003). However, the authors stated that imatinib could have been a confounding factor as 25% of patients taking imatinib were over age 75, while no patient taking other second-generation TKIs was over age 75 [8]. Advanced age is a well-established risk factor for serious illness and death regardless of the type of population studied [16–18, 21].

Conclusive evidence of the effectiveness of imatinib against COVID-19 must come from randomised clinical trials, and several are in progress around the world: Maryland, US (NCT04394416) [22], France (NCT04356495 and NCT04357613), Netherlands (EudraCT: 2020-001236-10) and Spain (NCT04346147).

The large sample of patients with CML who had access to multiple lines of TKI treatment is a strength of this study, as is the fact this group of patients form an open cohort in which prospective studies can be conducted. One limitation of this study is that several patients with CML lacked the funds to have SARS-CoV-2 diagnostic tests, which significantly limited the number of patients included in this analysis. Another limitation is that active SARS-CoV-2 infection was confirmed by RT-PCR in 23% of cases. Other cases were detected using antibody and antigen screening, which are not the diagnostic methods of choice due to their sensitivity and specificity limitations. Another factor to consider is that patients with a COVID-19 diagnosis were not treated at the CCERC, but were referred upon diagnosis to hospitals designated by the Honduran Secretary of Health. This process complicated the acquisition of relevant data on the therapeutic approaches to COVID-19.

## Conclusion

In a sample of 149 CML patients with at least one COVID-19 diagnostic test performed between June 2020 and March 2021, a 20% positivity was found. This sample was characterised by male predominance (56%), with a mean CML treatment duration of 6 years. Fifty-five percent of patients achieved major molecular response, with BCR-ABL ≤ 0.1% (IS). Twenty-one percent had comorbidities. There were no significant differences in these variables between patients diagnosed with COVID-19 and those who were not.

Seventy-four percent of patients with COVID-19 and CML had an asymptomatic or mild illness. Only one patient died, a man with significant comorbidities who recently began imatinib at a reduced dose. A higher incidence of COVID-19 than described in the literature was found, but also a lower fatality rate. No statistical association was found between disease severity and the type of TKI used or molecular response at the time of infection. The data from this study are consistent with the literature in that the prognosis for patients with CML infected with COVID-19 is better than for patients with other malignant haematological diseases. Moreover, these data support the current understanding that the main prognostic factors of COVID-19 are advanced age and chronic comorbidities. Determining the effectiveness of imatinib or other TKIs as potential COVID-19 treatments will require randomised clinical trials with adequate control groups.

## List of abbreviations

CML, Chronic myeloid leukaemia; CCERC, Emma Romero de Callejas Cancer Centre; Ph+, Philadelphia chromosome-positive; TKIs, Tyrosine kinase inhibitors.

## Conflicts of interest

The authors declare that they have no conflicts of interest with this research.

## Funding

None.

## Figures and Tables

**Table 1.** Distribution of patients with CML included in this analysis at COVID-19 screening.

Variable	Total (%)	COVID-19 *n* = 28	No-COVID-19 *n* = 112	*p*-value^a^
Sex				0.055
Male	83 (56)	22	61	
Female	66 (44)	9	57	
Age at diagnosis (mean)	45.9	50.6	44.7	0.0747^b^
TKI used at diagnosis				0.629
Imatinib	121 (81)	24	97	
Tasigna	19 (13)	4	15	
Sprycel	9 (6)	3	6	
TKI treatment duration (months)	75.7	65.7	78.4	0.223^a^
Molecular response				0.914
≤0.1% (IS)	82 (55)	16	66	
>0.1%–1% (IS)	14 (9)	4	11	
>1%–10% (IS)	21 (14)	4	17	
>10% (IS)	32 (22)	8	24	
Comorbidities				
Yes	23 (15)	8	15	0.073
No	126 (85)	23	103	

a Chi-squared test and Fisher’s exact test for qualitative variables, student’s *t*-test for parametric quantitative variables

b 95% confidence intervals for both groups overlap, losing statistical significance

**Table 2.** Frequency of COVID-19 signs and symptoms in patients with CML, *n* = 31.

Signs and symptoms	Frequency	Percentage
Fever	14	45.2
Muscle and joint pain	9	29.0
Cough	8	25.8
Dyspnoea	8	25.8
Odynophagia	4	12.9
Diarrhoea	4	12.9
Headache	4	12.9
Ageusia	4	12.9
Anosmia	3	9.7
Rash	1	3.2
Retro-ocular pain	1	3.2
Conjunctivitis	0	0.0

**Table 3.** Distribution of positive COVID-19 cases in patients with CML, by TKI received at viral diagnosis and disease severity.

TKI	COVID-19 severity				Total *n* (%)
	Asymptomatic	Mild	Moderate	Severe	
Imatinib	9	9	4	2	24 (77.4)
Nilotinib	2	1	0	1	3 (9.68)
Dasatinib	1	1	0	1	4 (12.9)
Total	12 (38.7)	11 (35.5)	4 (12.9)	4 (12.9)	31 (100)

**Table 4.** Distribution of COVID-19 disease severity in patients with CML, based on TKI received at detection.

COVID-19 severity	Type of TKI		OR	*p*-value	95% CI
	Imatinib	Other TKI			
Non-severe	18	5	0.833	0.8493	0.098–10.998
Severe	6	2			
Total	24	7			

**Table 5.** Distribution of COVID-19 case severity, by BCR-ABL molecular response at infection diagnosis.

BCR-ABL (IS)	COVID-19 severity				Total	*p*-value^a^
	Asymptomatic	Mild	Moderate	Severe		
<0.1%	10	4	1	1	16	0.083
>0.1%–10%	0	2	1	0	3	
>1%–10%	1	2	1	0	4	
>10%	1	3	1	3	8	
Total	12	11	4	4	31	

a Fisher’s exact test
